# HIV-1 Transactivator of Transcription (Tat) Co-operates With AP-1 Factors to Enhance *c-MYC* Transcription

**DOI:** 10.3389/fcell.2021.693706

**Published:** 2021-06-30

**Authors:** Leonardo Alves de Souza Rios, Lungile Mapekula, Nontlantla Mdletshe, Dharshnee Chetty, Shaheen Mowla

**Affiliations:** ^1^Division of Haematology, Department of Pathology, University of Cape Town, Cape Town, South Africa; ^2^Division of Anatomical Pathology, Department of Pathology, University of Cape Town, Cape Town, South Africa

**Keywords:** non-Hodgkin lymphoma, HIV-1, transactivator of transcription, *c-MYC*, AP-1

## Abstract

HIV-1 infection often leads to the development of co-morbidities including cancer. Burkitt lymphoma (BL) is one of the most over-represented non-Hodgkin lymphoma among HIV-infected individuals, and displays a highly aggressive phenotype in this population group, with comparatively poorer outcomes, despite these patients being on anti-retroviral therapy. Accumulating evidence indicates that the molecular pathogenesis of HIV-associated malignancies is unique, with components of the virus playing an active role in driving oncogenesis, and in order to improve patient prognosis and treatment, a better understanding of disease pathobiology and progression is needed. In this study, we found HIV-1 Tat to be localized within the tumor cells of BL patients, and enhanced expression of oncogenic c-MYC in these cells. Using luciferase reporter assays we show that HIV-1 Tat enhances the *c-MYC* gene promoter activity and that this is partially mediated via two AP-1 binding elements located at positions -1128 and -1375 bp, as revealed by mutagenesis experiments. We further demonstrate, using pull-down assays, that Tat can exist within a protein complex with the AP-1 factor JunB, and that this complex can bind these AP-1 sites within the *c-MYC* promoter, as shown by *in vivo* chromatin immunoprecipitation assays. Therefore, these findings show that in HIV-infected individuals, Tat infiltrates B-cells, where it can enhance the expression of oncogenic factors, which contributes toward the more aggressive disease phenotype observed in these patients.

## Introduction

People infected with HIV are at significantly higher risk of developing cancer. Although this risk has diminished since the introduction of Highly Active Antiretroviral Therapy (HAART), HIV-infected individuals remain over-represented within specific cancer groups, such as Kaposi’s sarcoma (KS), subtypes of non-Hodgkin lymphomas (NHL) and cervical cancer (Guiguet et al., 2009), when compared to the general population. Additionally, these individuals display more severe disease and have lower survival rates, often despite maintaining CD4^+^ T cell count at near-normal levels (Powles et al., 2009; Coghill et al., 2015). Factors attributable to this include chronic inflammation, and B-cell hyperactivation as a result of viral persistence, as well as co-infection with other oncogenic viruses (Robbins et al., 2015; Abudulai et al., 2016; Hart et al., 2018). In recent years, HIV and its viral components have been directly implicated in driving oncogenesis, and this is strongly evidenced in the development of KS, and more recently in B-cell derived malignancies (Xue et al., 2014; Cesarman et al., 2019; Santerre et al., 2019; Mdletshe et al., 2020). Although HIV-1 is known to only infect a subset of human cells, namely CD4^+^ cells, soluble HIV-1 proteins are detectable in the serum of HIV-infected individuals, and shown to invade and/or bind to the receptors of uninfected cells including B lymphocytes and endothelial cells (Lazzi et al., 2002; Eugenin et al., 2007; Xu et al., 2009; Lamers et al., 2010; Musinova et al., 2016; Anand et al., 2018). Upon entering bystander cells they interfere with host gene expression and other cellular processes, which are contributing factors to cellular transformation, and ultimately the development of HIV-associated cancers (Martorelli et al., 2015; Carroll et al., 2016; Santerre et al., 2019; Mdletshe et al., 2020).

The HIV-1 protein transactivator of transcription (Tat) has been shown to interfere with a variety of cellular processes in uninfected cells, and to also synergize with other oncogenic viruses such as Kaposi’s sarcoma-associated herpesvirus (KSHV), leading to enhanced angiogenesis and tumorigenesis in KS (Chen et al., 2009; Zhou et al., 2013; Yao et al., 2015; Yang et al., 2020). In HIV host cells, this small protein (86–101 amino acids long) plays a crucial role in HIV DNA transcription and survival. However, recent evidence shows a strong link between Tat and HIV-associated Burkitt Lymphoma (BL), where the viral protein was found to facilitate and enhance cellular events which promote this cancer. For instance, exposure of peripheral blood B-cells from healthy individuals to Tat led to remodeling of the B-cell genome, induction of oxidative DNA damage, and enhancement of expression of the *AICDA* gene, a promoter of c-*MYC/IGH* translocations (Germini et al., 2017; Sall et al., 2019). As more studies emerge, it has become evident that HIV-1 Tat can alter cellular events in complex and multiple ways, and one of the crucial events in the pathogenesis of a number of aggressive B-cell lymphomas is dysregulation of *c-MYC*. Due to its highly oncogenic nature, the expression of this transcription factor is tightly regulated at both the transcriptional and translational levels in normal cells. c-MYC is essential for early B-cell development in the bone marrow but barely detectable in Germinal Centre (GC) B cells (Nguyen et al., 2017). Conversely, *c-MYC* translocation and detection has become essential in the clinical diagnosis and prognosis of aggressive B-cell lymphomas including BL.

In this study, we investigated the presence of HIV-1 Tat within BL tumor cells, and its ability to influence the expression of c-MYC, a transcription factor that plays a primary oncogenic role in a majority of cancers, including BL (Ramiro et al., 2004; Takizawa et al., 2008; Greisman et al., 2012). Using immunohistochemistry we demonstrate the presence of Tat in the tumor cells of BL patients. We further show that c-MYC expression is enhanced in BL cells where Tat is expressed, and we demonstrate that this enhancement is partially as a result of transcriptional regulation of the *c-MYC* promoter, through the collaboration of Tat with AP-1 factors, and via AP-1 sites located within the *c-MYC* promoter.

## Materials and Methods

### Cell Lines and Culturing Conditions

The human Burkitt lymphoma cell line Ramos was obtained from ATCC (Manassas, VA) and the BL41 cell line was a kind donation from Professor Dave from Duke University, United States. Both cell lines were grown in Roswell Park Memorial Institute (RPMI-1640) media (Sigma-Aldrich, United States) supplemented with 10% FBS (Sigma-Aldrich, United States) and 1% penicillin/streptomycin (Sigma-Aldrich, United States) or 20% FBS post electroporation. Cells were incubated in a 5% CO_2_ humidified incubator at 37°C. The human HT1080 cell line (fibrosarcoma cell line) was used as a host cell line when performing luciferase assays due to its high transfection efficiency and was cultured in Dulbecco’s Modified Eagle’s Medium (DMEM) (Sigma-Aldrich, United States) supplemented with 10% foetal bovine serum (FBS) (Invitrogen, United States) and 1% penicillin/streptomycin (Sigma-Aldrich, United States).

### Immunohistochemistry

Twelve (12) HIV positive BL and one HIV negative DLBCL formalin fixed paraffin embedded (FFPE) samples were retrieved from the National Health Laboratory Services Anatomical Pathology patient archive, at the Groote Schuur Academic Hospital in Cape Town, South Africa. FFPE 5 μm tissue sections were incubated at 60°C for 15 min on a heating plate and allowed to cool at RT for 5 min. Next, the tissue sections were de-waxed in xylene and rehydrated through graded alcohol. The EnVision Flex Mini Kit, High pH (Link) (K8023; Dako, United States) was used for the Immunohistochemical staining according to manufacturer recommendations. Briefly, sections were incubated in 2.5% BSA/TBS blocking solution for 15 min at RT. Thereafter, the HIV negative DLBCL and HIV positive BL tumor sections were incubated with anti-HIV-1 Tat antibody (ab43014; Abcam, United Kingdom; 1:100 in 2.5% BSA/TBS) overnight at 4°C and rinsed in wash buffer for 5 min. Sections were incubated in 100 μL of HRP secondary antibody (K8023; Dako, United States) for 1 h at RT followed by another wash for 5 min. Color development was achieved by incubating the tissue sections in Elution substrate buffer containing DAB Chromagen solution (K8023; Dako, United States) at RT for 10 min. Thereafter, the slides were counterstained with Mayer’s Hematoxylin and Scott’s solution, dehydrated and cleared with xylene and mounted with Entellan (107960; Merck, Germany). Sections incubated with only 2.5% BSA/TBS were included as negative controls. All imaging was performed on a Zeiss Axioskop 2 upright microscope with AxioVision 4 software.

### Electroporation of BL Cells

BL cell lines were counted at the log phase of growth and 4 × 10^6^ cells were transferred to 0.4 cm electroporation cuvettes at a total volume of 450 μL RPMI-1640 media without supplementation. 15 μg of either pcDNA-Tat or pcDNA3.1 empty control (both plasmids were kindly donated by Professor Mitra from the National Center for Cell Sciences in India) was added to the cuvettes and electroporated using the Gene Pulser Xcell system (Bio-Rad, United States) with the following conditions: exponential wave, 0.28 kV and 950 μF. Cells were incubated on ice for 10 min followed by gentle resuspension and transfer to prewarmed RPMI-1640 media supplemented with 20% FBS (Sigma-Aldrich, United States) and 1% penicillin/streptomycin (Sigma-Aldrich, United States). Cells were incubated for 24-48 h before protein was harvested for western blot analysis.

### Protein Isolation and Western Blotting

Total protein was isolated from electroporated cells using 2x Laemmli buffer (0.125 mM Tris-HCL pH 6.8, 4% SDS, 10% β-mercaptoethanol, 20% glycerol, and 0.005% of bromophenol blue) and boiled for 5 min at 95°C. Protein was separated using 12% SDS-PAGE gels and transferred to nitrocellulose membranes (Bio-Rad) using the Mini-PROTEAN 3 casting apparatus (Bio-Rad). The following antibodies were used to detect target proteins: primary antibodies were anti-c-MYC (SC-764, Santa Cruz Biotechnology, United States; 1:1,000), anti-HIV-1 Tat (NIH AIDS reagent program (2A4.1 4373; 1:1,000), anti-JunB (SC-8051, Santa Cruz Biotechnology, United States; 1:2,000) and anti-p38 (M0800, Sigma-Aldrich, United States; 1:5,000). Secondary antibodies were Goat Anti Rabbit (H + L) HRP conjugate (170-6515, Bio-Rad, United States; 1:5,000) and Goat Anti Mouse (H + L) HRP conjugate (170-6516, Bio-Rad, United States; 1:5,000). Densitometric analysis of the signal intensity of bands was done using the ImageJ software (NIH, United States).

### Promoter Sequence Analysis and Generation of Deletion Constructs

The WT *c-MYC* promoter (−2324 to + 537 bp) cloned within the pGL3-Basic luciferase reporter vector (gift of Professor Dai from the University of California, United States) was analyzed using PROMO (Algorithmics and Genetics Group, Universitat Politecnica de Catalunya)^1^ to identify potential TF binding sites. Deletion constructs of the *c-MYC* promoter were designed to gradually remove promoter regions using primers with incorporated restriction enzyme sites. PCR was performed with the MyTaq^TM^ DNA polymerase kit (Bioline, United States) using forward and reverse primers (with incorporated restriction enzyme (RE) sites) and the plasmid containing the full-length WT promoter of the *c-MYC* gene as the template. Forward primers were designed to incorporate a *Sac*I RE site: DF1- 5′-GGGAGCAGAGCTCTCATGTGTGGG-3′, DF2- 5′-GGCGCAAAGAGCTCTTGTCTCTTCTG-3′, DF3- 5′ -CTAGAGCGAGCTCGCTCGGCTGCC-3′ and the reverse primer was designed to incorporate a *Hin*dIII RE site: R-5′-CCAAGCTTGCTACTCTGCAGGTCG-3′.

### Site-Directed Mutagenesis

Forward and reverse primers containing the desired DNA base pair modifications (underlined) were generated to disrupt AP-1 binding sites. AP-1 site 1 and 2 refer to the AP-1 TFBS at positions -1128 and -1375 bp relative to the TSS, respectively, AP-1 Mut1 F- 5′-CACAAGG GTCTCTGCCGTAGTCCCCGGCTCGGTCCACAAG-3′, AP-1 Mut1 R- 5′-CTTGTGGACCGAGCCGGGGACTACGGCAGAGA CCCTTGTG-3′, AP-1 Mut2 F-5′-CAGAAAAAATTGCGTAGT AGTGAACTAGGAAATTAATGCCTGGAAGGC-3′, AP-1 Mut 2 R-5′-GCCTTCCAGGCATTAATTTCCTAGTTCACTACTACG CAATTTTTTCTG-3′, The KAPA HiFi HotStart ReadyMix system (Roche, Germany) was used to generate the mutated plasmids from the template pGL3-c-MYC-WT plasmid Sanger Sequencing was performed to confirm successful mutations (Inqaba Biotec).

### Transfections and Dual-Luciferase Reporter Assays

Dual-luciferase reporter assays were conducted in HT1080 cells seeded at a density of 5 × 10^4^ cells/mL in six-well cell culture dishes. Four hundred nanograms of each luciferase reporter, full-length as well as deletions of the *c-MYC* promoter, were co-transfected using X-tremeGene^TM^ HP transfection reagent with up to 500 nanograms of pcDNA-Tat or the corresponding empty vector. The pRL-TK driving the expression of a Renilla reporter was used as an internal control for transfection efficiency. Thirty hours post-transfection, whole-cell extracts were assayed for firefly and Renilla luciferase activity using the dual-luciferase reporter assay system (Promega, United States). Luciferase activities were measured using the GloMax^®^-Multi + Luminescence Module (Promega, United States). Firefly luciferase values were normalized to the Renilla luciferase activity and expressed relative to empty vector control. Experiments were performed at least in triplicate.

### Co-immunoprecipitation

HT1080 cells (5 × 10^4/^mL) were plated in six-well cell culture plates. After 24 h, the cells were co-transfected with 500 ng of pcDNA-Tat and 500 ng pCMV-JunB plasmids with the XtremeGene-HP reagent (Sigma-Aldrich, United States). Cells were harvested after 24 h and lysed using RIPA buffer (50 mM TRIS HCL pH8, 150 mM NaCl, 1% Triton X, 0.5% sodium deoxycholate and 0.1% SDS) combined with 1X complete^TM^, Mini, EDTA-free, Protease Inhibitor (Roche, Switzerland). Protein samples were precleared with 50 μL protein A agarose beads (Roche, Switzerland) for 3 h at 4°C. The Pierce^®^ BCA Protein Assay kit (Thermo Fisher Scientific, United States) was used to quantify the protein and 200 μg of protein was used per pulldown. Two micrograms of primary antibody (anti-JunB (SC-8051, Santa Cruz Biotechnology, United States), anti-HIV-1 Tat (NIH AIDS reagent program (2A4.1 4373) or ab43014, Abcam, United Kingdom) and negative control IgG (170-6515, Bio-Rad, United States) were each added to individual tubes containing the 200 μg of protein and incubated overnight at 4°C. Fifty microliters protein A agarose beads were added to the protein-antibody complexes and incubated for 4 h at 4°C. Beads were collected by centrifugation at 2,000 rpm and washed twice in ice-cold 1x PBS containing protease inhibitors. Beads were pelleted and washed twice in ice-cold 1x PBS followed by resuspension in 2x Laemmli Blue, boiled for 5 min at 95°C followed by SDS-PAGE separation and western blot analysis. Experiments were performed at least in triplicate.

### Chromatin Immunoprecipitation

HT1080 cells (5 × 10^4/^mL) were plated in six-well cell culture plate. After 24 h, cells were transfected with 500 nanograms of both pcDNA-Tat and pCMV-JunB plasmids using the XtremeGene-HP reagent. Cells were cross-linked with 1% formaldehyde, quenched with 125 mM glycine followed by lysis and sonication to obtain chromatin fragments between 300 and 500 bp in length. Sonicated DNA was cleared with protein A agarose beads for 4 h at 4°C followed by overnight incubation in primary antibodies, anti-JunB (SC-8051, Santa Cruz Biotechnology, United States), anti-HIV-1 Tat (ab43014, Abcam, United Kingdom) and negative control IgG (170-6515, Bio-Rad, United States) at 4°C. DNA enrichment was analyzed through qRT-PCR using the following *c-MYC* promoter-specific primers: Primer Set 1 F 5′-GGAATTAAACGTCCGGTTTGTC-3′, Primer Set 1 R 5′-GGCAAGTGGAGAGCTTGTG-3′, Primer Set 2 F 5′-GCAACTAGCTAAGTCGAAGCG-3′, Primer Set 2 R 5′-GGCAAGTGGAGAGCTTGTG-3′. Cycling conditions were as follows: initial denaturation at 95°C for 3 min, 40 cycles including denaturation at 95°C for 5 s, combined annealing/extension at 55°C for 20 s and a final extension at 55°C for 8 min. Experiments were performed at least in triplicate.

### Statistical Analysis

Student’s *t*-tests and one-way analysis of variance (ANOVA) tests were used. Significance was accepted at *p* < 0.05. Statistical significance was determined using the GraphPad Prism version 8.4.1 for Windows, GraphPad Software, San Diego, California, United States.

## Results

### HIV-1 Tat Is Detectable in the Tumors of HIV Positive BL Patients, and Tat-Expression in BL Cells Leads to Enhanced c-MYC Expression

**FIGURE 1:**
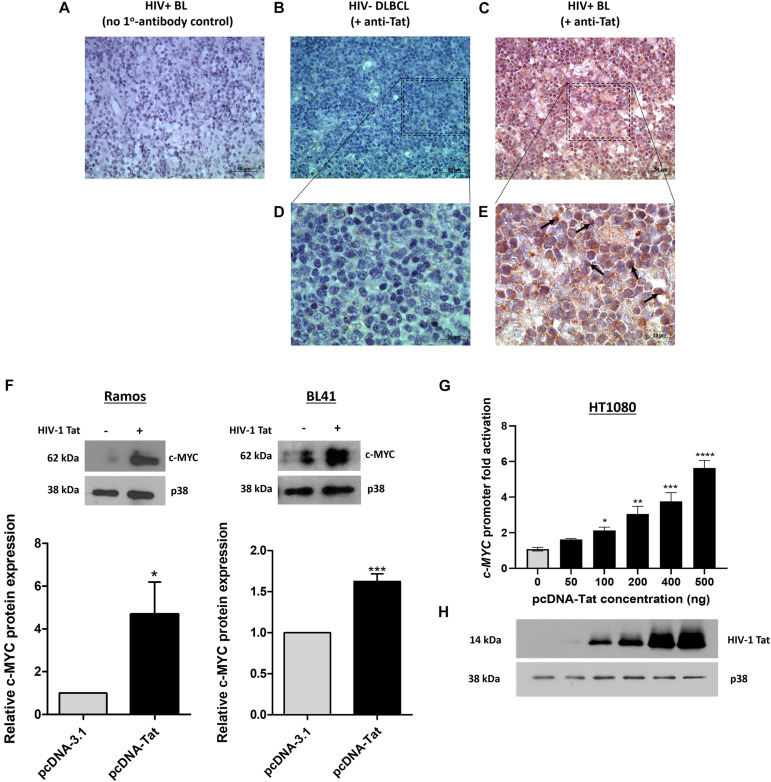
HIV-1 Tat is detected in BL tumor tissue and elevates c-MYC expression at the transcriptional and translational levels. **(A–E)** Immunohistochemical detection of Tat protein in FFPE tumor samples from lymphoma patients. An HIV positive BL incubated without primary antibody (replaced with buffer only) **(A)** and an HIV negative DLBCL incubated with primary Tat antibody (ab43014, Abcam, United Kingdom) as used for the HIV positive BL tumor samples **(B)**, were used as controls. **(C)** IHC for Tat protein expression in HIV positive BL tumor tissue. Positive staining is indicated by deposition of DAB (brown) and Hematoxylin was used as counterstain (blue/purple). Distinct nuclear Tat expression (marked by black arrows in **E**). Images **(A–C)** were taken at 400X magnification and **(D,E)** at 1000X. Scale bar in **(A–C)** represents 50 and 20 μm in **(D,E)**. **(F)** Western blotting of total proteins isolated from Ramos (Left) and BL41 (Right) cells electroporated with pcDNA-Tat expression vector or pcDNA-Empty control using anti-c-MYC (SC-764, Santa Cruz Biotechnology, United States) antibody. Anti-p38 (M0800, Sigma-Aldrich, United States) was used as a loading control. Bar graphs represent densitometric analysis of western blots (ImageJ) and error bars represent standard deviation (SD). **(G)** Dual-Luciferase Reporter assay shows a significant dose-dependent increase in *c-MYC* promoter activity with increasing HIV-1 Tat concentration, relative to the empty-vector control which has been set at 1. The following fold increases were obtained for the indicated pcDNA-Tat concentrations: 50 ng: 1.5 ± 0.15; 100 ng: 2 ± 0.03; 200 ng: 2.86 ± 0.2; 400 ng: 3.5 ± 0.2 and 500 ng: 5.3 ± 0.02. Error bars represent SD. **(H)** Western blot showing increasing expression of HIV- 1 Tat in transfected HT1080 cells in G, with p38 as a loading control. Statistical analysis was done using GraphPad PRISM 8 with Student’s *t*-test and one-way ANOVA, significance indicates ^∗^(*p* < 0.05), ^∗∗^(*p* < 0.01) ^∗∗∗^(*p* < 0.001), ^****^(*p* < 0.0001). The results are representative of at least three separate repeats.

An early study demonstrated the presence of Tat using immunohistochemistry (IHC), within the tumor cells of HIV-associated B-cell lymphoma (Lazzi et al., 2002). We found Tat to be localized within all 12 of the formalin-fixed paraffin embedded (FFPE) tumor samples from HIV-associated BL patients whom we tested (Table 1) and a representation of the IHC result is shown in (Figures 1C,E). This was shown to be specific, as we were unable to detect Tat within the tumor cells of an HIV negative DLBCL patient (Figures 1B,D), and HIV positive BL tumors where no primary Tat antibody was included (Figure 1A). BL arising in HIV uninfected individuals are rare and were not available within our archives to use as a negative control. Higher magnification and careful analysis revealed that some cells displayed distinct nuclear staining (Figure 1E—indicated by black arrows). Control samples included BL samples incubated with BSA only (without Tat antibody) (Figure 1A), as well as an HIV negative DLBCL sample probed using an antibody against Tat, to show that Tat was specific to tumors from HIV infected patients only (Figures 1B,D). To determine if Tat expression within BL cells correlates with an increase in c-MYC expression, western blot analysis was performed in the BL cell lines Ramos and BL41 in which Tat was ectopically expressed using electroporation. BL cells electroporated with a plasmid that allows for constitutive expression of Tat had significantly elevated c-MYC protein levels compared to the control cells which were transfected with an empty vector (Figure 1F). Furthermore, using luciferase reporter assays, we could demonstrate that a wild-type (WT) promoter of *c-MYC* (−2324 to + 537 bp) was significantly activated by up to 5.65 (± 0.42) −fold, in a dose-dependent manner, in the presence of increasing amounts of Tat (Figure 1G). Due to the extremely low efficiency at which lymphocytic cells take up foreign DNA, Tat protein could not be detected in the BL cells post-electroporation, however, we could verify electroporation using a GFP-tagged vector (data not shown), but more importantly, copious amounts of the protein could be detected by western blotting in the HT1080 host cells used in the reporter assays, which were transfected with the same plasmid (Figure 1H).

**TABLE 1:** Clinical features of the HIV-BL cohort investigated in this study.

Patients #, Sex (age in years)	HAART duration (months)	Plasma HIV RNA (copies/mL)	CD4^+^ T cells (× 10^6/^L)	FISH [MYC-IGH t(8;14) (q24;q32)] translocation	% B cell nuclei with MYC-IGH translocation
1, M (30)	NRA	Undetectable	337	+	40
2, M (26)	NRA	77	221	+	100
3, F (53)	NRA	12 000	324	+	55
4, M (39)	NRA	NRA	NRA	+	55
5, F (30)	NRA	NRA	NRA	+	92
6, F (25)	0	NRA	84	NRA	
7, F (34)	0	NRA	NRA	NRA	
8, F (32)	1	120	NRA	NRA	
9, F (44)	0	NRA	249	−	0
10, F (50)	NRA	NRA	160	NRA	
11, M (33)	NRA	NRA	700	NRA	
12, M (59)	NRA	Undetectable	NRA	NRA	

*HAART, Highly active antiretroviral therapy; FISH, Fluorescence in situ hybridization; NRA, No record available.*

### The *c-MYC* Promoter Activation by HIV-1 Tat Is Partially Mediated by AP-1 Binding Elements

**FIGURE 2:**
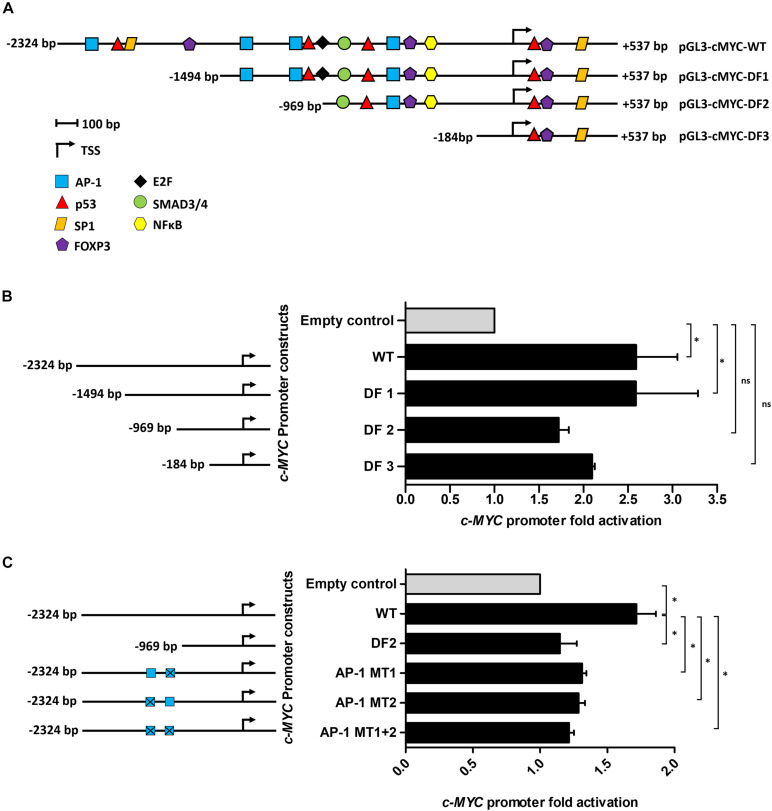
The AP-1 sites located at positions -1128 and -1375 bp partially mediate the effect of HIV-1 Tat on the *c-MYC* promoter. **(A)** The WT *c-MYC* promoter (-2324 to + 537 bp) construct was analyzed for TFBS using PROMO. Sites are represented as different colored shapes which are labeled in the key and the transcription start site (TSS) is denoted by a black arrow. Shortened promoter constructs are labeled accordingly on the right side of the diagram (pGL3-cMYC-DF1, pGL3-cMYC-DF2 and pGL3-cMYC-DF3). **(B)** Luciferase reporter assays comparing the fold activation of the c-MYC WT promoter to the deletion constructs (DF1-DF3) in the presence of HIV-1 Tat. **(C)** Luciferase assays comparing fold activation in the presence of HIV-1 Tat between the WT promoter, DF2 and AP-1 MT1, AP-1 MT2 and AP-1 MT1 + 2 constructs. Fold activation was obtained by comparing the relative luciferase units (RLU) of the pcDNA-Tat transfected groups to their empty transfected controls, which were set to 1. Statistical analysis was done using one-way ANOVA in GraphPad PRISM 8. Significance ^∗^(*p* < 0.05) and ns (not significant). Error bars represent SD. The results are representative of at least three separate repeats.

Using the online software PROMO, a comprehensive analysis of the WT *c-MYC* promoter was performed to identify putative transcription factor binding sites (TFBS). The dissimilarity rate was set at 15% to not dismiss potential binding sites. Transcription factors (TFs) with previously reported associations, directly or indirectly, with Tat, or which have been implicated in BL pathobiology were annotated and mapped (Figure 2A). Luciferase reporter assays using the WT promoter and a series of promoter deletion constructs (Figure 2A) identified the region -1494 to -969 bp (pGL3-cMYC-DF2 construct) of the *c-MYC* promoter to play an important role in Tat-mediated activation. The activity of the WT promoter was significantly enhanced in the presence of HIV-1 Tat (2.6 ± 0.46-fold), and this remained unchanged with the loss of promoter region -2324 to -1494 bp (DF1). However, no significant increase in promoter activity was observed within DF2 and DF3, relative to the empty control, when promoter regions -1494 to -969 bp and -969 to -184 bp were lost, respectively. Activator protein 1 (AP-1) had been previously shown to enhance the HIV-1 Tat-mediated transcription of the viral LTR (van der Sluis et al., 2014). Furthermore, several reports have linked gene alteration, via enhancement or alteration of AP-1 binding to promoter regions, in the presence of HIV-1 Tat, indicating that this viral protein has a cooperating relationship with AP-1 factors in altering cellular gene expression (Lim and Garzino-Demo, 2000; Hidalgo-Estévez et al., 2006; Blanco et al., 2008). We identified two AP-1 binding sites within the -1494 to -969 bp region of the promoter (AP-1 site 1 at position -1128 bp and AP-1 site 2 at position -1375 bp) and both had favorable dissimilarity scores indicating that these sites were potentially functional *in vivo*, with site 2 being previously reported to induce *c-MYC* transcription upon Platelet-Derived Growth Factor (PDGF) receptor stimulation (Iavarone et al., 2003). Indeed, mutation of AP-1 site 1 and 2 led to significant losses of ∼20 and 25% promoter activity, respectively, in comparison to the WT promoter, while a construct carrying mutations on both sites led to an overall loss in promoter activity of ∼29% (Figures 2B,C). These results indicated that HIV-1 Tat was able to enhance the activity of the *c-MYC* promoter via AP-1 binding sites, and potentially through the activity of AP-1 factors.

### HIV-1 Tat and JunB Proteins Form a Complex and Can Bind AP-1 Sites *in vivo*

**FIGURE 3:**
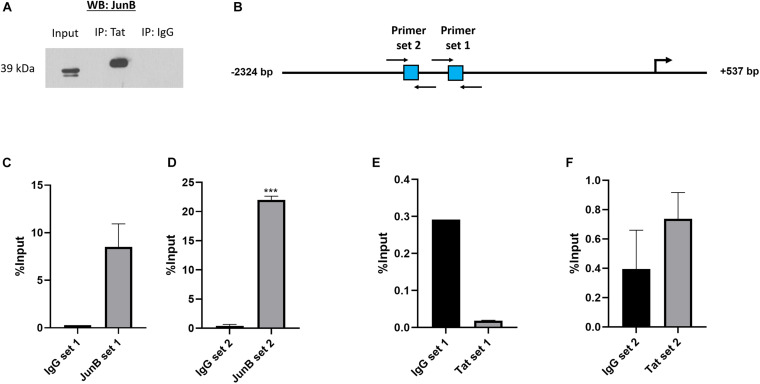
HIV-1 Tat and JunB form protein to protein associations and occupy the *c-MYC* promoter at two AP-1 binding sites. HT1080 cells were transfected with both 500 ng HIV-1 Tat expression vector (pcDNA-Tat) and 500 ng JunB expression vector (pCMV-JunB). **(A)** The cell lysate was incubated with anti-HIV-1 Tat antibody (ab43014, Abcam, United Kingdom) and pulled down using protein A agarose beads (Roche, Germany). Western blot was carried out using an anti-JunB antibody (Santa Cruz, United States) The goat-anti-rabbit secondary antibody (Bio-Rad, United States) was used as a negative control (lane 3). **(B)** Diagrammatic representation of the *c-MYC* promoter depicting the location of the two AP-1 sites in square blocks and the positions of the forward and reverse primers used for the ChIP assay are denoted by black arrows. **(C–F)** Bar graphs show qRT-PCR data using immunoprecipitated DNA obtained from ChIP with either anti-JunB, anti-HIV-1 Tat or anti-IgG (negative control) antibodies and amplified with the primers depicted in the diagram, error bars represent SD. Statistical significance determined using Student’s *t*-test in GraphPad PRISM 8, ^∗∗∗^(*p* < 0.001). The graphs are representative of at least three separate repeats.

Previous studies have shown that HIV-1 Tat can form complexes with cellular proteins including transcription factors. For instance, Tat was found to form a complex with NFAT and c-Jun at an NFAT/AP-1 composite site (Hidalgo-Estévez et al., 2006). There is evidence that Tat can alter cellular gene expression both through direct binding of promoters, as well as through complexing with transcription factors (Lim and Garzino-Demo, 2000; Carvallo et al., 2017). Using an *in vitro* co-immunoprecipitation assay we found that Tat can form a complex with the AP-1 factor JunB, which is a major component of AP-1 complexes, and previously reported to associate with Tat (Kumar et al., 1998; Hidalgo-Estévez et al., 2006; van der Sluis et al., 2014). Using an HIV-1 Tat specific antibody, protein complexes were pulled down in Tat-expressing cells and subjected to western blotting using an antibody specific to JunB and the result shows that JunB and Tat co-exist within a protein complex (Figure 3A). The reverse experiment, i.e., pull-down using an antibody specific to JunB could not be achieved because the antibody is not compatible with IP assays (data not shown). Nevertheless, we found confidence in this result by the fact that two independent anti-Tat antibodies were able to successfully pull down JunB in separate assays. Further confirmatory evidence of the Tat/JunB/c-MYC axis was obtained using *in vivo* chromatin-immunoprecipitation (ChIP) assays, using two primer sets that spanned AP-1 sites 1 and 2 (Figure 3B). The results showed that both AP-1 sites were enriched when chromatin was pulled down with JunB antibodies (Figures 3C,D), with a significantly pronounced enrichment at AP-1 site 2 (Figure 3D). Pull down with HIV-1 Tat antibodies showed no enrichment at site 1 (Figure 3E), while enrichment was found at site 2, albeit lower levels, and although not statistically significant, this result was reproducible (Figure 3F). These results indicate that HIV-1 Tat and the AP-1 factor JunB exist within a protein complex that binds with *c-MYC* promoter at AP-1 binding elements.

## Discussion

Infection with HIV-1 is associated with the development of multiple cancers. Amongst these, the highly aggressive B-cell lymphoma BL is particularly over-represented among the HIV positive population, of which Sub-Saharan Africa has one of the highest incidences in the world (Abayomi et al., 2011; Phillips and Opie, 2018). Recent studies have brought attention to the oncogenic potential of HIV-1 and its associated proteins in promoting these cancers, and the molecular mechanism driving these phenomena is still poorly defined. An early study by Lazzi et al. (2002) demonstrated that Tat protein is detected in AIDS-related B-cell lymphomas with noticeable intracellular localization identified through IHC, and the hypothesis is that soluble Tat protein circulating in the serum of HIV-infected individuals can become internalized by non-host cells, where they act to promote oncogenic events. To the best of our knowledge, no other study has reported the same HIV-1 Tat expression and localization as reported by Lazzi et al. (2002) and we thus sought to determine if the same phenomenon occurred within BL tumor cells from a cohort of our HIV-BL patients. We observed distinct and specific nuclear Tat protein expression in the majority of stained tissue sections, which suggests that this viral protein may play a role in gene expression alterations via direct and/or indirect DNA binding. We found that expression of the highly oncogenic transcription factor c-MYC was enhanced, at both the transcriptional and translational levels, in the presence of HIV-1 Tat. The translocation of *c-MYC* to the *IgH* locus is a pathologic feature of BL, however, hardly any studies have assessed c-MYC expression from the intact *c-MYC* allele within the tumor cells. This is particularly relevant within the context of an HIV-positive background, given the more aggressive nature of the disease in HIV-positive patients, even those receiving ART. Notably, our findings are supported by work done by Germini et al. (2017), where the authors showed that B cells exposed extracellularly to recombinant Tat protein had increased *c-MYC* mRNA expression.

This study demonstrates that Tat collaborated with the cellular transcription factor JunB to bind the *c-MYC* promoter and drive expression. The affinity of Tat for promoter regions of host genes has been described in the context of T-cell infection. Genome-wide association studies have revealed that Tat binds to a variety of regions within the human T cell genome (Marban et al., 2011; Dhamija et al., 2015; Reeder et al., 2015). Notably, in Jurkat cells (acute T cell leukemia cell line), HIV-1 Tat was found to preferentially occupy sites within gene promoters and 5′-UTRs and importantly, these associations were conserved in RNA-Seq data showing that Tat not only binds to but also alters the expressions of host genes, specifically genes involved in the cellular immune response. Other studies have shown that Tat forms protein-protein interactions with host TFs, including AP-1 factors, and augments their binding to promoters of host genes (Southgate and Green, 1991; Lim and Garzino-Demo, 2000; Blanco et al., 2008; Kim et al., 2010). While two AP-1 binding elements within the *c-MYC* promoter were identified to be mediators of Tat-driven expression, these were partial, and thus further studies are needed to identify alternative sites or mechanisms driving this phenomenon. Indirect mechanisms could be via the activation of upstream pathways such as the JNK pathway, shown to influence c-MYC expression, and this pathway has been shown to be activated by Tat in both B and T cells (Kumar et al., 1998; Ju et al., 2012). Additionally, B cells exposed extracellularly to recombinant Tat have shown enhanced ROS production which could potentially induce the JNK pathway and lead to increased AP-1 phosphorylation (El-Amine et al., 2018). Interestingly, a positive feedback loop between c-MYC and AP-1 factors has been proposed in BL, with increased c-MYC levels leading to increased AP-1 phosphorylation and binding to the Igκ Ei and E3′ enhancers, potentially increasing expression of translocated *c-MYC* in *Ig*κ*/c-MYC*-BL and possibly that of the unaffected allele on chromosome 8 (Ding et al., 2020). The increased transcriptional activity of the *c-MYC* gene due to Tat, coupled with its increased localization to the *IgH* locus and aberrant AID activity, could collectively be the reason for the enhancement of cell proliferation, genetic aberrations, and a more aggressive disease phenotype in HIV positive people.

## Data Availability Statement

The original contributions presented in the study are included in the article/supplementary material, further inquiries can be directed to the corresponding author/s.

## Ethics Statement

The studies involving human participants were reviewed and approved by University of Cape Town, Faculty of Health Sciences, Human Research Ethics Committee. The patients/participants provided their written informed consent to participate in this study.

## Author Contributions

LA performed most of the experimental work and data analysis and wrote the first draft of the manuscript. NM performed the luciferase assays in Figure 1G. LM performed the IHC experiment and generated Figure 1A, and contributed to the first draft of the manuscript. SM conceptualized and supervised the research, performed some data analyses, and wrote and edited the manuscript. DC facilitated access to the FFPE tumor tissues, coordinated the sectioning, and assisted in the analysis of the IHC results. All authors read and approved the final manuscript.

## Conflict of Interest

The authors declare that the research was conducted in the absence of any commercial or financial relationships that could be construed as a potential conflict of interest.

## Footnotes

1 http://alggen.lsi.upc.es/cgibin/prom-v3/promo/promoinit.cgi?dirDB=TF_8.3

## References

[B1] Abayomi E. A. Somers A. Grewal R. Sissolak G. Bassa F. Maartens D. (2011). Impact of the HIV epidemic and anti-retroviral treatment policy on lymphoma incidence and subtypes seen in the western cape of South Africa, 2002–2009: preliminary findings of the tygerberg lymphoma study group. *Transfus. Apher. Sci.* 44 161–166. 10.1016/j.transci.2011.01.007 21402310 PMC3899789

[B2] Abudulai L. N. Fernandez S. Corscadden K. Hunter M. Kirkham L.-A. S. Post J. J. (2016). Chronic HIV-1 infection induces B-cell dysfunction that is incompletely resolved by long-term antiretroviral therapy. *J. Acquir. Immune Defic. Syndr.* 71 381–389. 10.1097/QAI.0000000000000869 26914910

[B3] Anand A. R. Rachel G. Parthasarathy D. (2018). HIV proteins and endothelial dysfunction: implications in cardiovascular disease. *Front. Cardiovasc. Med.* 5:185. 10.3389/fcvm.2018.00185 30619892 PMC6305718

[B4] Blanco A. Álvarez S. Fresno M. Muñoz-Fernández M. Á (2008). Extracellular HIV-tat induces cyclooxygenase-2 in glial cells through activation of nuclear factor of activated T cells. *J. Immunol.* 180 530–540. 10.4049/jimmunol.180.1.530 18097055

[B5] Carroll V. A. Lafferty M. K. Marchionni L. Bryant J. L. Gallo R. C. Garzino-Demo A. (2016). Expression of HIV-1 matrix protein p17 and association with B-cell lymphoma in HIV-1 transgenic mice. *Proc. Natl. Acad. Sci. U.S.A.* 113 13168–13173. 10.1073/pnas.1615258113 27799525 PMC5135339

[B6] Carvallo L. Lopez L. Fajardo J. E. Jaureguiberry-Bravo M. Fiser A. Berman J. W. (2017). HIV-Tat regulates macrophage gene expression in the context of neuroAIDS. *PLoS One* 12:e0179882. 10.1371/journal.pone.0179882 28640909 PMC5481010

[B7] Cesarman E. Damania B. Krown S. E. Martin J. Bower M. Whitby D. (2019). Kaposi sarcoma. *Nat. Rev. Dis. Prim.* 5:9. 10.1038/s41572-019-0060-9 30705286 PMC6685213

[B8] Chen X. Cheng L. Jia X. Zeng Y. Yao S. Lv Z. (2009). Human immunodeficiency virus type 1 Tat accelerates Kaposi sarcoma-associated herpesvirus Kaposin A-mediated tumorigenesis of transformed fibroblasts in vitro as well as in nude and immunocompetent mice. *Neoplasia* 11 1272–1284. 10.1593/neo.09494 20019835 PMC2794508

[B9] Coghill A. E. Shiels M. S. Suneja G. Engels E. A. (2015). Elevated cancer-specific mortality among HIV-infected patients in the United States. *J. Clin. Oncol.* 33 2376–2383. 10.1200/JCO.2014.59.5967 26077242 PMC4500831

[B10] Dhamija N. Choudhary D. Ladha J. S. Pillai B. Mitra D. (2015). Tat predominantly associates with host promoter elements in HIV-1-infected T-cells - regulatory basis of transcriptional repression of c-Rel. *FEBS J.* 282 595–610. 10.1111/febs.13168 25472883

[B11] Ding X. Wang X. Zhu X. Zhang J. Zhu Y. Shao X. (2020). JNK/AP1 pathway regulates MYC expression and BCR signaling through Ig enhancers in burkitt lymphoma cells. *J. Cancer* 11 610–618. 10.7150/jca.34055 31942184 PMC6959055

[B12] El-Amine R. Germini D. Zakharova V. V. Tsfasman T. Sheval E. V. Louzada R. A. N. (2018). HIV-1 tat protein induces DNA damage in human peripheral blood B-lymphocytes via mitochondrial ROS production. *Redox Biol.* 15 97–108. 10.1016/j.redox.2017.11.024 29220699 PMC5725280

[B13] Eugenin E. A. King J. E. Nath A. Calderon T. M. Zukin R. S. Bennett M. V. L. (2007). HIV-tat induces formation of an LRP-PSD-95-NMDAR-nNOS complex that promotes apoptosis in neurons and astrocytes. *Proc. Natl. Acad. Sci. U.S.A.* 104 3438–3443. 10.1073/pnas.0611699104 17360663 PMC1805607

[B14] Germini D. Tsfasman T. Klibi M. El-Amine R. Pichugin A. Iarovaia O. V. (2017). HIV Tat induces a prolonged MYC relocalization next to IGH in circulating B-cells. *Leukemia* 31 2515–2522. 10.1038/leu.2017.106 28360415

[B15] Greisman H. A. Lu Z. Tsai A. G. Greiner T. C. Yi H. S. Lieber M. R. (2012). IgH partner breakpoint sequences provide evidence that AID initiates t(11;14) and t(8;14) chromosomal breaks in mantle cell and Burkitt lymphomas. *Blood* 120 2864–2867. 10.1182/blood-2012-02-412791 22915650 PMC3466967

[B16] Guiguet M. Boué F. Cadranel J. Lang J.-M. Rosenthal E. Costagliola D. (2009). Effect of immunodeficiency, HIV viral load, and antiretroviral therapy on the risk of individual malignancies (FHDH-ANRS CO4): a prospective cohort study. *Lancet Oncol.* 10 1152–1159. 10.1016/S1470-2045(09)70282-7 19818686

[B17] Hart B. B. Nordell A. D. Okulicz J. F. Palfreeman A. Horban A. Kedem E. (2018). Inflammation-related morbidity and mortality among HIV-positive adults: how extensive is it? *J. Acquir. Immune Defic. Syndr.* 77 1–7. 10.1097/QAI.0000000000001554 28991883 PMC5720921

[B18] Hidalgo-Estévez A. M. González E. Punzón C. Fresno M. (2006). Human immunodeficiency virus type 1 tat increases cooperation between AP-1 and NFAT transcription factors in T cells. *J. Gen. Virol.* 87 1603–1612. 10.1099/vir.0.81637-0 16690925

[B19] Iavarone C. Catania A. Marinissen M. J. Visconti R. Acunzo M. Tarantino C. (2003). The platelet-derived growth factor controls c-myc expression through a JNK- and AP-1-dependent signaling pathway. *J. Biol. Chem.* 278 50024–50030. 10.1074/jbc.M308617200 14523011

[B20] Ju S. M. Goh A. R. Kwon D. J. Youn G. S. Kwon H. J. Bae Y. S. (2012). Extracellular HIV-1 tat induces human beta-defensin-2 production via NF-kappaB/AP-1 dependent pathways in human B cells. *Mol. Cells* 33 335–341. 10.1007/s10059-012-2287-0 22450687 PMC3887796

[B21] Kim N. Kukkonen S. Gupta S. Aldovini A. (2010). Association of Tat with promoters of PTEN and PP2A subunits is key to transcriptional activation of apoptotic pathways in HIV-infected CD4+ T cells. *PLoS Pathog.* 6:e1001103. 10.1371/journal.ppat.1001103 20862322 PMC2940756

[B22] Kumar L. J. Ghosh M. R. Tucker P. W. (1998). HIV-tat protein activates c-jun N-terminal kinase and activator protein-1. *J. Immunol.* 161: 776. 9670954

[B23] Lamers S. L. Fogel G. B. Huysentruyt L. C. McGrath M. S. (2010). HIV-1 nef protein visits B-cells via macrophage nanotubes: a mechanism for AIDS-related lymphoma pathogenesis? *Curr. HIV Res.* 8 638–640. 10.2174/157016210794088209 21067513 PMC3471533

[B24] Lazzi S. Bellan C. De Falco G. Cinti C. Ferrari F. Nyongo A. (2002). Expression of RB2/p130 tumor-suppressor gene in AIDS-related non-Hodgkin’s lymphomas: implications for disease pathogenesis. *Hum. Pathol.* 33 723–731. 10.1053/hupa.2002.125372 12196924

[B25] Lim S. P. Garzino-Demo A. (2000). The human immunodeficiency virus type 1 tat protein up-regulates the promoter activity of the beta-chemokine monocyte chemoattractant protein 1 in the human astrocytoma cell line U-87 MG: role of SP-1, AP-1, and NF-κB consensus sites. *J. Virol.* 74 1632–1640. 10.1128/JVI.74.4.1632-1640.2000 10644332 PMC111637

[B26] Marban C. Su T. Ferrari R. Li B. Vatakis D. Pellegrini M. (2011). Genome-wide binding map of the HIV-1 Tat protein to the human genome. *PLoS One* 6:e26894. 10.1371/journal.pone.0026894 22073215 PMC3208564

[B27] Martorelli D. Muraro E. Mastorci K. Dal Col J. Faè D. A. Furlan C. (2015). A natural HIV p17 protein variant up-regulates the LMP-1 EBV oncoprotein and promotes the growth of EBV-infected B-lymphocytes: implications for EBV-driven lymphomagenesis in the HIV setting. *Int. J. Cancer* 137 1374–1385. 10.1002/ijc.29494 25704763

[B28] Mdletshe N. Nel A. Shires K. Mowla S. (2020). HIV Nef enhances the expression of oncogenic c-MYC and activation-induced cytidine deaminase in Burkitt lymphoma cells, promoting genomic instability. *Infect. Agent. Cancer* 15:54. 10.1186/s13027-020-00320-9

[B29] Musinova Y. R. Sheval E. V. Dib C. Germini D. Vassetzky Y. S. (2016). Functional roles of HIV-1 Tat protein in the nucleus. *Cell. Mol. Life Sci.* 73 589–601. 10.1007/s00018-015-2077-x 26507246 PMC11108392

[B30] Nguyen L. Papenhausen P. Shao H. (2017). The Role of c-MYC in B-Cell Lymphomas: Diagnostic and Molecular Aspects. *Genes (Basel).* 8:116. 10.3390/genes8040116 28379189 PMC5406863

[B31] Phillips L. Opie J. (2018). The utility of bone marrow sampling in the diagnosis and staging of lymphoma in South Africa. *Int. J. Lab. Hematol.* 40 276–283. 10.1111/ijlh.12782 29427399

[B32] Powles T. Robinson D. Stebbing J. Shamash J. Nelson M. Gazzard B. (2009). Highly active antiretroviral therapy and the incidence of non-AIDS-defining cancers in people with HIV infection. *J. Clin. Oncol.* 27 884–890. 10.1200/JCO.2008.19.6626 19114688

[B33] Ramiro A. R. Jankovic M. Eisenreich T. Difilippantonio S. Chen-Kiang S. Muramatsu M. (2004). AID is required for c-myc/IgH chromosome translocations in vivo. *Cell* 118 431–438. 10.1016/j.cell.2004.08.006 15315756

[B34] Reeder J. E. Kwak Y. T. McNamara R. P. Forst C. V. D’Orso I. (2015). HIV Tat controls RNA Polymerase II and the epigenetic landscape to transcriptionally reprogram target immune cells. *Elife* 4:e08955. 10.7554/eLife.08955 26488441 PMC4733046

[B35] Robbins H. A. Pfeiffer R. M. Shiels M. S. Li J. Hall H. I. Engels E. A. (2015). Excess cancers among HIV-infected people in the United States. *J. Natl. Cancer Inst.* 107:dju503. 10.1093/jnci/dju503 25663691 PMC4334816

[B36] Sall F. B. El Amine R. Markozashvili D. Tsfasman T. Oksenhendler E. Lipinski M. (2019). HIV-1 Tat protein induces aberrant activation of AICDA in human B-lymphocytes from peripheral blood. *J. Cell. Physiol.* 234 15678–15685. 10.1002/jcp.28219 30701532

[B37] Santerre M. Chatila W. Wang Y. Mukerjee R. Sawaya B. E. (2019). HIV-1 Nef promotes cell proliferation and microRNA dysregulation in lung cells. *Cell Cycle* 18 130–142. 10.1080/15384101.2018.1557487 30563405 PMC6343720

[B38] Southgate C. D. Green M. R. (1991). The HIV-1 Tat protein activates transcription from an upstream DNA- binding site: implications for Tat function. *Genes Dev.* 5 2496–2507. 1752440 10.1101/gad.5.12b.2496

[B39] Takizawa M. Tolarová H. Li Z. Dubois W. Lim S. Callen E. (2008). AID expression levels determine the extent of cMyc oncogenic translocations and the incidence of B cell tumor development. *J. Exp. Med.* 205 1949–1957. 10.1084/jem.20081007 18678733 PMC2526190

[B40] van der Sluis R. M. Derking R. Breidel S. Speijer D. Berkhout B. Jeeninga R. E. (2014). Interplay between viral Tat protein and c-Jun transcription factor in controlling LTR promoter activity in different human immunodeficiency virus type I subtypes. *J. Gen. Virol.* 95 968–979. 10.1099/vir.0.059642-0 24447950

[B41] Xu W. Santini P. A. Sullivan J. S. He B. Shan M. Ball S. C. (2009). HIV-1 evades virus-specific IgG2 and IgA responses by targeting systemic and intestinal B cells via long-range intercellular conduits. *Nat. Immunol.* 10 1008–1017. 10.1038/ni.1753 19648924 PMC2784687

[B42] Xue M. Yao S. Hu M. Li W. Hao T. Zhou F. (2014). HIV-1 Nef and KSHV oncogene K1 synergistically promote angiogenesis by inducing cellular miR-718 to regulate the PTEN/AKT/mTOR signaling pathway. *Nucleic Acids Res.* 42 9862–9879. 10.1093/nar/gku583 25104021 PMC4150761

[B43] Yang W.-S. Lin T.-Y. Chang L. Yeh W. W. Huang S.-C. Chen T.-Y. (2020). HIV-1 tat interacts with a Kaposi’s sarcoma-associated herpesvirus reactivation-upregulated antiangiogenic long noncoding RNA, LINC00313, and antagonizes its function. *J. Virol.* 94 e1280–e1219. 10.1128/jvi.01280-19 31723026 PMC7000985

[B44] Yao S. Hu M. Hao T. Li W. Xue X. Xue M. (2015). MiRNA-891a-5p mediates HIV-1 tat and KSHV Orf-K1 synergistic induction of angiogenesis by activating NF-κB signaling. *Nucleic Acids Res.* 43 9362–9378. 10.1093/nar/gkv988 26446987 PMC4627096

[B45] Zhou F. Xue M. Qin D. Zhu X. Wang C. Zhu J. (2013). HIV-1 tat promotes Kaposi’s Sarcoma-Associated Herpesvirus (KSHV) vIL-6-induced angiogenesis and tumorigenesis by regulating PI3K/PTEN/AKT/GSK-3β signaling pathway. *PLoS One* 8:e53145. 10.1371/journal.pone.0053145 23301033 PMC3534639

